# Effect of Increasing the Foot Area on the Load-Velocity Relationship of the Underwater Dolphin Kick

**DOI:** 10.5114/jhk/189796

**Published:** 2024-12-06

**Authors:** Shuxin Wang, Yixiao Zhao, Xiaotong Chen, Yupeng Shen

**Affiliations:** 1 School of Physical Education and Sports Science, South China Normal University, Guangzhou, Guangdong, China.; 2 College of Physical Education and Health, Guangdong Polytechnic Normal University, Guangzhou, Guangdong, China.

**Keywords:** swimming, fins, load-velocity profile, semi-tethered

## Abstract

The objective of this study was to evaluate the impact of augmenting the foot area (by wearing fins) on the load-velocity relationship of the underwater dolphin kick (UDK) and to investigate the optimal loading zone of resistance training for the UDK. Seventeen swimmers underwent a semi-tethered swimming test and a 15-m maximum swim velocity test, both with and without fins (FINS and WF, respectively). The study revealed that the UDK's load-velocity relationship, when using semi-tethered swimming, displayed a robust linear correlation (R^2^ = 0.88 ± 0.15). The FINS condition enhanced the optimization of the load-velocity relationship, resulting in a substantial rightward shift (R^2^, AIC, BIC optimized by 15%–65%) and elevating the UDK velocity by 10%–22% across seven load levels. The effective load level rose from 57 N to 69 N (R = 0.70–0.85, p < 0.05); however, the FINS condition altered the original UDK technique, leading to a 7% decrease in the stroke rate (SR) and a 19% increase in stroke length (SL). Consequently, wearing fins modified the load-velocity relationship of the UDK and augmented the power output level. We recommend that athletes use semi-traction swimming to improve UDK performance with a maximum load of no more than 57 N or a velocity of no less than 73% of maximum velocity; wearing fins allows this range to be extended to 69 N and 71% of maximum velocity.

## Introduction

The underwater dolphin kick (UDK) is a swimming technique executed after the start and a turn of a swimming race. In this technique, the athlete extends their arms overhead, maintains a streamlined body position, and relies on up-and-down kicking to propel their body forward. Some studies have demonstrated that increasing the UDK velocity can enhance performance after the start and a turn, ultimately improving overall swimming race performance. Consequently, optimizing UDK performance has increasingly captured researchers' attention (Fischer and Kibele, 2016; Ruiz-Navarro et al., 2024; Veiga et al., 2024).

An athlete's capacity to generate and sustain the highest forward velocity of the UDK primarily depends on minimizing resistance and maximizing propulsive force. This necessitates having sufficient muscle strength and the ability to transfer it effectively to the water. Thus, researchers are keenly interested in evaluating athletes' strength performance in water. Load-velocity profiling (LVP) is an effective method for characterizing muscle mechanical force, and utilizing LVP can assist coaches and athletes in assessing the theoretical maximum force, power, and velocity capacities that muscles can produce. This information can be employed to establish individualized training prescriptions (Bielec et al,. 2013, 2021; Bobbert et al., 2016; Demirkan et al., 2023; García-Ramos et al., 2016; Morin and Samozino, 2016). To date, LVP has been more commonly applied to muscle strength assessment in land-based athletes (Cross et al., 2016). Due to water's unstable nature, few studies have incorporated the load-velocity relationship in swimming research. Currently, semi-tethered swimming is considered the optimal method for conducting LVP in water. In semi-tethered swimming, additional loads are provided by mechanical-construction equipment or electronically-constructed load devices, and athletes are connected to these devices by a non-elastic rope to evaluate propulsive power or load-velocity relationship changes in water through multiple incremental load trials. Early studies indicated that athletes' swimming velocity exhibited a strong linear fit with the extra load, R^2^ = 0.99 ± 0.01 (Olstad et al., 2020). Recent studies with high reliability of load-velocity relationships established in butterfly and freestyle events revealed a significant association between these variables and swimming performance; the horizontal F_0_, P_max_, and V_0_ were nearly perfectly correlated with 5–40 m sprint times (Baena-Raya et al., 2021). Researchers concluded that the load-velocity relationship could be developed as an effective tool for the indirect assessment of the swimming propulsion load and velocity.

The increasingly prevalent in-water load-velocity profiling has emerged as an effective tool for evaluating swimming propulsion and velocity. However, there are still many aspects to examine concerning the UDK. First, there are differences in the active drag between swimming styles (Lopes et al., 2022), and UDK experiences less drag compared to other surface competitive swimming styles. The specificity of the technique makes it challenging to directly apply the load-velocity relationship from other swimming styles to the UDK. Secondly, load-velocity profiling is an effective tool for indirectly measuring the propulsive force-velocity relationship. One study has demonstrated that using fins can increase the propulsive area of the foot and displace a larger volume of water, thereby enhancing propulsive efficiency and force (Matos et al., 2013). This finding suggests that changes in the foot area may influence the load-velocity relationship for the UDK, but there is a dearth of research evaluating this phenomenon. Furthermore, load-velocity profiling can serve as a common method not only for specialized testing, but also for water-specific resistance training (Girold et al., 2006, 2007). Previous studies have observed that additional loading results in a significant decrease in swimming velocity and stroke length. Therefore, it remains to be confirmed through research whether increasing the foot area (using fins) can effectively mitigate this adverse effect. Based on the aforementioned considerations, the objective of this study was to establish a load-velocity profile for the UDK using semi-tethered swimming and to investigate whether augmenting the foot area (with fins) would acutely affect the varibles of the load-velocity relationship and to investigate the optimal loading zone of resistance training for the UDK.

## Methods

### Participants

Seventeen university swimmers who had received long-term systematic training (male: 13; female: 4; age: 21.7 ± 1.3 years) participated in this experiment (Table 1). All participants had average training experience of eight years and were in good physical condition without any injuries. None of the participants had stayed up late or consumed alcohol 24 h before the experiment. The study was conducted in accordance with the Declaration of Helsinki and approved by the Institutional Review Board of the South China Normal University (protocol code: SCNU-SPT-2022-101; approval date: 22 November 2022). All swimmers provided their written informed consent prior to participation.

**Table 1:** Basic information of participants.

	Male (n = 13)	Female (n = 4)
Height (cm)	177.6 ± 5.8	176.4 ± 3.9
Weight (kg)	71.3 ± 9.3	68.9 ± 9.0
Time of 15-m WF-UDK (s)	9.15 ± 0.75	11.94 ± 0.97
Time of 15-m Fins-UDK (s)	7.49 ± 0.52	9.55 ± 1.62
Tail area increment (%)	44.1% ± 13.4%	43.8% ± 7.1%

### Design and Procedures

A cross-sectional study design was employed. The test was divided into two parts: wearing fins (FINS) and without fins (WF). Participants selected the day's test by lottery and were required to complete the remaining part of the test within a week from the end of the day. The tests were conducted in a standard 50-m swimming pool (50 m x 21 m), with water and air temperatures maintained at 28°C and 27°C, respectively. All participants wore swimsuits that complied with FINA regulations. Before conducting the in-water test, researchers collected anthropometric data from participants. Swimmers performed their own pre-competition warm-up (of approximately 30 min) before the test to closely resemble regular competition conditions. Following the warm-up, participants underwent an active recovery period of 10–20 min (Neiva et al., 2013). During this time, a lottery was conducted to determine the type of a test to be performed that day, i.e., wearing fins or without fins. If the swimmer drew the test for wearing fins, they were also required to select the appropriate size of fins. Two sizes (size D and size F) of the same type of fins were provided to participants based on their foot size. On average, the fins provided participants with additional 44% of the foot area. In the study, participants were instructed to perform two 15-m underwater dolphin kicks (UDKs) with maximum effort. The best performance out of the two attempts was selected for inclusion in the final analysis. Following a 20-min rest period (Neiva et al., 2013), participants completed seven UDK semi-tethered swimming tests (load-velocity relationship tests) at maximum effort. In the seven tests, the loads used were 21 N, 33 N, 45 N, 57 N, 69 N, 81 N, and 93 N, denoted as L1 to L7. Participants were given a 5-min rest interval between each test.

According to a previous study, lower loads and fewer tests result in a more accurate load-velocity relationship (Olstad et al., 2022). Therefore, the aforementioned test protocol with a maximum load of 92 N (10 kg) was considered adequate.

### Measurement of the Foot Area

**Figure 1:**
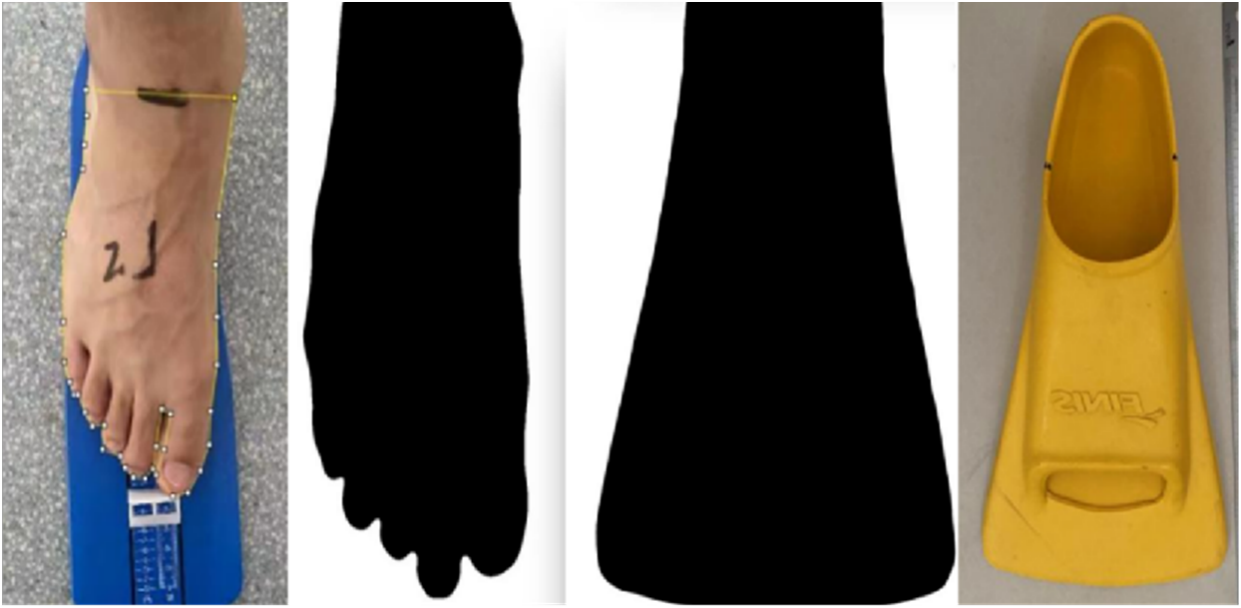
Tail wing area calculation

Zoomer Gold fins (Finis, USA; Figure 1) were employed in this study. These fins, made of rubber with small blades and moderate stiffness, are favored by athletes and coaches. The area of the participant's feet was measured using a combination of tools, including a digital camera (iPhone 13 Pro, Apple, USA), Image J 1.48 software (NIH, USA), and a Foot Ruler from China (Figure 1). Image J is a Java-based image processing program developed by the National Institutes of Health. It features an open image processing architecture that can be used for image processing and quantification through plug-ins and digital cameras. Considering that the forefoot provides the primary propulsive force during swimming, we calculated only the forefoot area of each participant from the lower end of the tibia to the toe and the area of the fins. The difference was divided by the forefoot area to obtain the percentage of the propulsive area increment (tail area increment %).

### Fifteen-Meter Underwater Dolphin Kick Performance

**Figure 2:**
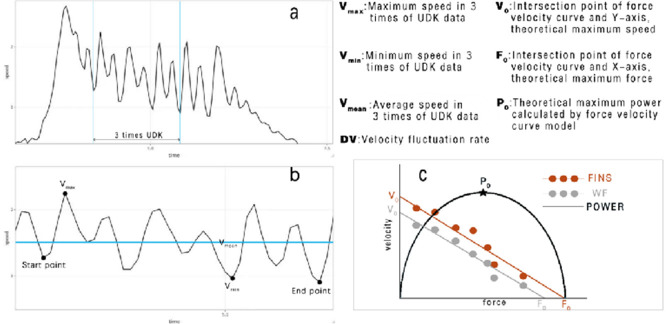
An example of the intra-cycle velocity, load-velocity relationship and power curves. a: original image; b: shows how to manually select three complete action cycles on a velocity-time curve; c: load-power image.

A swim velocity meter (SWIMSPORTEC, Germany) was employed to record velocity data in all tests, including the 15-m test and the semi-tethered swim test. The meter was attached to the participant's hip joint via a thin, non-elastic wire and measured the instantaneous velocity data produced during the test at a sampling frequency of 31 Hz. The velocity-time plot (Figure 2a) was generated by smoothing the raw data filtered by the gauges using the fourth-order Butterworth method. To prevent any external factors from affecting the data, researchers manually excluded the initial and final movements of the curve. They then selected three complete movements from the middle of the curve (Figure 2b) to calculate various variables such as average velocity, maximum velocity, minimum velocity, a velocity fluctuation rate, a stroke rate, and stroke length (V_mean_, V_max_, V_min_, DV, SR, SL, respectively). In this study, one kick cycle started at the highest toe vertical coordinate and ended with the next highest peak thereafter. Each kick cycle was divided into two kick phases: a downward kick and an upward kick (Atkison et al., 2014). The downward kick started from the highest toe vertical coordinate to the lowest point, and an upward kick started from the lowest point to the highest point (Matsuura et al., 2020). The SR and SL were respectively defined as the ratio of frequency to time and the ratio of distance to frequency.

**Figure 3:**
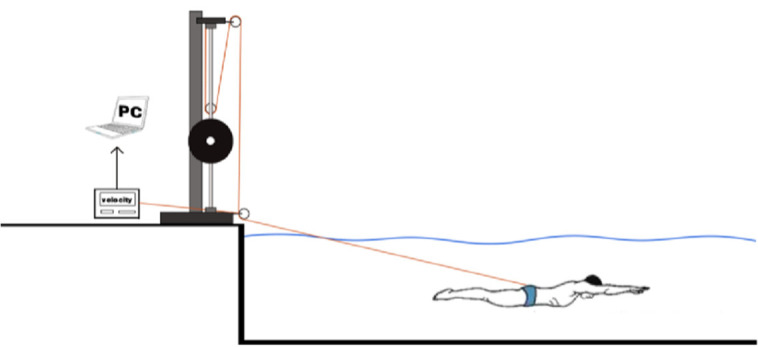
Semi-tethered swimming test.

### Load-Velocity Profiling

In the semi-tethered swimming test (the load-velocity relationship test), we utilized a modified version of the Smith machine that provided an additional load for the swimmers. This Smith machine had two pulleys fixed to the top and the bottom, as well as a dynamic pulley suspended above the load. To exclude interference of the test results by pushing off the pool wall, a soft, non-elastic cord with unloaded length of 2 m was used to connect the load to a belt located at the participant's hip joint. The Smith machines were calibrated using loads ranging from 5 kg to 60 kg. All loads were placed in the same position as during testing. A regression equation, with an R^2^ value of 0.9879, was utilized to account for the impact of the pulley system on loads, including mechanical savings and friction. The equation took into account the force value provided by the mechanics sensor (x) and the actual force value (y):


y=1.3902x+43.464

To ensure accurate velocity calculations, we selected three consecutive action cycles located in the middle of the velocity-time curve. This approach is consistent with the 15-m UDK performance test and helps avoid potential over or underestimation of velocity. The average velocity (V_mean_) during the three cycles was plotted as functions of the corresponding loads. Linear regression lines were generated for each load-velocity plot. The coefficient of determination (R^2^), theoretical maximum velocity (V_0_), and the theoretical maximum load (F_0_) were calculated for each participant using the regression line. F_0_ was also expressed as a percentage of body mass (RF_0_) and the slope was determined based on the load-velocity relationship regression line. Figure 2c displays a sample load-velocity plot for a single participant.

### Statistical Analysis

The study data are described using mean and standard deviation. For each participant, a load-velocity regression model was established using the minimum squared error method. The coefficient of determination (R^2^) and standard error estimate (SEE) were reported to evaluate the goodness of fit of the regression model. The Akaike information criterion (AIC) and the Bayesian information criterion (BIC) were reported to evaluate the goodness of fit of the regression model under the without fins (WF) and wearing fins (FINS) conditions. The smaller the AIC and the BIC, the better the regression model. In order to determine the difference between the load-velocity relationship profile variables, a paired *t*-test was conducted for the UDK while wearing fins (FINS) and for the UDK without fins (WF). Cohen's *d* was used to determine the magnitude of the differential effect size with the following criteria: if |Cohen's *d*| < 0.20, the effect size was considered small, if it was between 0.20 and 0.50, it was considered moderate, and if it was > 0.5, it was considered large. Correlation analysis was performed using Pearson's correlation's. A correlation coefficient (R) of 0.1 or less was considered very low, while a value between 0.1 and 0.3 was considered low. A correlation coefficient between 0.3 and 0.5 was considered moderate, between 0.5 and 0.7 was considered high, and a coefficient of 0.7 or higher was considered very high. The level of statistical significance was set at *p* < 0.05.

We also used the following formulas to calculate the percentage decrease in UDK velocity at different loads without wearing fins (VLT) and the percentage increase in velocity after increasing the propulsive surface area (ΔVLT) to evaluate the effects of the load and the fin area on velocity (WFL0: average velocity of the 15-m UDK without fins; WFLn: average velocity of the UDK without fins at a certain load level; FINSLn: average velocity of the UDK with an increased foot area at a certain load level; n = load level):


$$\begin{array}{l} \text{VLT}=\frac{\text{WFL}0-\text{WFLn}}{\text{WFL}0}\text{*}100\text{\%}\\ \text{VLT}=\frac{\text{FINSLn}-\text{WFLn}}{\text{WFLn}}\text{*}100\text{\%} \end{array}$$

## Results

### Effect of Increasing the Foot Propulsion Area on the Performance of the 15-m UDK

Under the FINS condition, V_mean_, SL, and V_max_ increased by 16%, 19%, 9% and 9%, respectively, with large to extremely large effect size (*d* = −1.509 to −0.871). In contrast, swimmers maintained similar V_min_ and SR when wearing the fins as they did without them (Table 2).

**Table 2:** Effects of increasing the foot area on variables in the 15-m UDK test

	unit	M ± SD		t	df	Cohen's *d*
		WF	FINS			
V_mean_	m/s	1.46 ± 0.19	1.7 ± 0.15	−6.223	16	−1.509***
V_max_	m/s	2.1 ± 0.25	2.3 ± 0.25	−3.59	16	−0.871**
V_min_	m/s	0.92 ± 0.23	1.01 ± 0.16	−1.187	16	−0.288
SR	cycle/s	2.8 ± 0.66	2.6 ± 0.30	1.501	16	0.364
SL	m/cycle	0.54 ± 0.096	0.64 ± 0.08	−4.651	16	−1.128***
DV	%	3.7 ± 0.86	3.6 ± 0.75	0.392	16	0.095

p < 0.05*; p < 0.01**; p < 0.001***

WF: Underwater dolphin kick without fins; FINS: Underwater dolphin kick with fins; V_mean_: Centroid velocity; V_max_: Maximum velocity; V_min_: Minimum velocity; SR: Stroke rate; SL: Stroke length; DV: Velocity fluctuation rate

### Effect of Increasing the Foot Area on the UDK Load-Velocity Relationship

**Figure 4:**
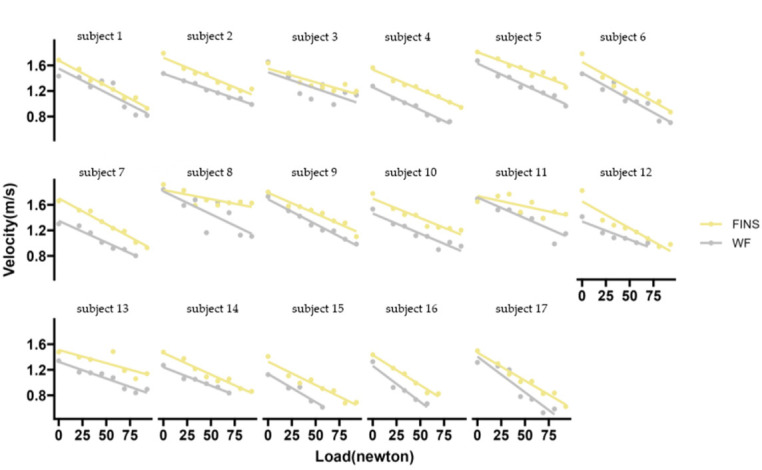
Load-velocity relationship of all participants. FINS representatives with fins; WF representative for without fins; M for males and W for females

Figure 4 shows the LV relationships for all participants considering both foot areas, which proved to be very good for the UDK regardless of whether swimmers wore fins or not. Therefore, all variables (V_0_, F_0_, P_0_) in the LV relationship showed a significant positive correlation with the maximum velocity in the 15-m UDK test (Table 3). Wearing fins produced a significant improvement in the LV relationship in the UDK, with a 15% increase in R^2^ and a 65% decrease in the AIC and the BIC (Table 4). At the same time, wearing fins increased V_0_, P_0_, and F_0_ by 14%, 12%, and 26%, respectively. Thus, the LV relationship produced a significant rightward shift when wearing fins. In contrast, for the LV slope, there was only a small enhancement when wearing the fins.

**Table 3:** Correlation of 15-m velocity test performance with LV relationship profile variables

	unit	R	
		WF	FINS
V_0_	m/s	0.675**	0.657**
slope		0.509*	0.532*
F_0_	N	0.724**	0.636**
RF_0_	N/kg	0.538*	0.517*
P_0_	W	0.87***	0.669**

* p < 0.05, ** p <± 0.01, *** p < 0.001

V_0_: Theoretical maximum average velocity; slope: The slope of the LV relationship;

F_0_: The biggest force in theory; RF_0_: Relative F_0_,F_0_/weight; P_0_: Theoretical maximum power

**Table 4:** Effect of increasing the foot area on the load-velocity relationship profile parameters.

	M ± SD		t	df	Cohen's *d*
	WF	FINS			
R^2^	0.819 ± 0.196	0.941 ± 0.037	−2.816	16	−0.683*
SEE	0.077 ± 0.051	0.062 ± 0.027	1.537	16	0.373
AIC	−17 ± 11	−28 ± 11	5.056	16	1.226***
BIC	−17 ± 11	−26 ± 10	4.461	16	1.082***
slope	−0.0064 ± 0.0026	−0.0059 ± 0.0015	−0.931	16	−0.226
V_0_ *(m/s)*	1.4 ± 0.18	1.6 ± 0.17	−5.515	16	−1.338***
F_0_ *(N)*	255 ± 103	285 ± 79	−1.667	16	−0.404
P_0_ *(W)*	91 ± 39	115 ± 41	−4.159	16	−1.009***
RF_0_ *(N/kg)*	3.6 ± 1.3	4.0 ± 0.80	−1.608	16	−0.39

* p < 0.05;** p < 0.01;*** p < 0.001

R^2^: Coefficient of determination; SEE: Standard Error Estimate; AIC: Akaike information criterion; BIC: Bayesian information criterion

### Improvement of the Payload Interval by Increasing the Foot Area

After gradually increasing the load, a significant decrease in V_mean_ was observed, especially at the L6 level where the maximum decrease exceeded 37%, yet this situation improved significantly when wearing the fins (Table 5). However, the maximum velocity of the 15-m UDK was only associated with V_mean_ for load levels L1 to L4, but the load in question was expanded from L1 to L5 under the FINS condition (Table 5).

**Table 5:** Decrease in the average velocity of UDK on L1–L7 load; correlation between the velocity of UDK in semi-tethered swimming test on L1~L7 load and the velocity in the 15-m test.

	V15	L1	L2	L3	L4	L5	L6	L7
VLT		13%	16%	26%	27%	31%	35%	34%
ΔVLT	13%	16%	12%	21%	17%	17%	22%	10%
WF		0.89***	0.86***	0.70**	0.88***	−0.079	−0.42	−0.11
FINS	0.76***	0.87***	0.79***	0.83***	0.78***	0.85***	0.20	0.11

p < 0.05*; p < 0.01**; p < 0.001***

V15: Average velocity in the fifteen-meter UDK test; L1–L7: This corresponds to the external load of 21N, 33N, 45N, 57N, 69N, 81N, and 93N, respectively. VLT: Percentage of velocity decline under different loads, VLT = (L0 − L1) / L0. ΔVLT: Percentage of velocity improvement after increasing tail area. ΔVLT = (FINSL0 − WFL0) /WFL0

## Discussion

The purpose of this study was to assess the load-velocity relationship (LV) of the underwater dolphin kick (UDK) considering two different foot propulsion areas (WF: without fins; FINS: wearing fins) and to explore the reasonable loading intervals for UDK semi-tethered swimming in both cases.

It was observed in the study that the LV relationship clearly showed a highly linear relationship in either WF or FINS (R^2^ = 0.88 ± 0.117). This supports the validity of using semi-tethered swimming to assess the LV relationship for the UDK and is consistent with previous studies on front crawl, butterfly, and backstroke (Gonjo et al., 2020; Olstad et al., 2020, 2022). A moderate to large correlation was shown between the 15-m UDK variables (V15) and the LV relationship profile variables (V_0_, F_0_, P_0_, and slope), which indicates that the LV relationship profile variables are good predictors of UDK performance.

An important finding of this study is that increasing the foot propulsive area induces beneficial changes in the LV relationship profile variables of the UDK. Compared with the WF condition, the LV relationship of FINS had better fit superiority. This indicates that increasing the foot propulsive area can optimize the LV relationship of the UDK. From the images of LV, the LV relationship of FINS undergoes a significant shift to the right, and the LV relationship profile variables (V_0_, F_0_, P_0_) are enhanced to a larger extent than under the WF condition. This shows substantial benefits of increasing the foot propulsion area on UDK's swimming velocity as well as propulsion force and power. Variables (V_mean_, V_max_) obtained from the 15-m UDK maximum velocity test showed a similar trend, i.e., under the FINS condition, swimming velocity was higher when compared to the WF condition. Previous studies have also shown that cetaceans possess a larger tail area (propulsion area) and therefore exhibit higher swimming velocities (Loebbecke et al., 2009) and propulsive power levels (Fish, 1993) than humans. However, contrary to expectations, F_0_ obtained by swimmers wearing fins was only marginally higher (*d* = 0.40) compared to those without fins. This suggests that wearing fins may not result in a greater benefit to swimmers in terms of propulsive force. Zamparo et al. (2002) found that a non-linear relationship between the increase in the propulsive area and the increase in propulsive power, with the athlete wearing fins increasing the propulsive area of the foot by approximately 360%, but the propulsive power output increased only by 36%. Therefore, a possible explanation is that increasing the foot area of the athlete causes a direct change in propulsive efficiency rather than triggers a linear increase in the athlete's propulsive force. In a recent study, the slope was found to have a direct relationship with the active drag (AD) that swimmers endured in the water, with the greater slope being associated with greater AD (Gonjo et al., 2022). This is similar to the phenomenon observed in the present study. According to the Bernoulli's principle, the velocity of movement of objects in fluids is positively correlated with resistance; the faster the velocity, the greater the resistance. Therefore, athletes produced a slightly greater slope in the LV relationship test under the FINS when compared to the WF condition.

In the current study, the value of building the LV relationship to develop resistance training programs is gradually being affirmed by practitioners, although the effectiveness of applying the LV relationship to aquatic resistance training in swimmers is still controversial. Previous research has shown that when using semi-tethered swimming, the extra load in the particular water environment may have some negative impact on the athlete's swimming technique, causing a significant decrease in stroke length. That study inferred that this would affect the effectiveness of in-water resistance training (Dominguez-Castells and Arellano, 2012). In this regard, we focused on the correlation between the velocity obtained in the 15-m UDK test (V15) and the swimming velocity of athletes subjected to different loads. Our study demonstrates that regardless of whether athletes increased their foot propulsion area, UDK swimming velocity only showed a significant positive correlation with lower load levels, i.e., lower external loads were more beneficial for UDK training (Table 5). In a previous study on front crawl, it was pointed out that when the external load was less than or equal to 4 kg, the trend of the instantaneous velocity of front crawl was not significantly different from that of the instantaneous velocity of front crawl without additional loads; when the external load continued to increase, the instantaneous velocity curve of front crawl produced a significant change (Dominguez-Castells et al., 2012). In that study, authors showed that a significant decrease in stroke length was produced in the front crawl due to excessive external loading, thus affecting the instantaneous velocity change in the front crawl (Dominguez-Castells et al., 2012). Some researchers even suggest that when performing semi-tethered swimming, athletes or coaches should prefer these loads that are more correlated with maximal velocity (Soncin et al., 2021). The lesson learned from land sports is that developing the relative muscle capacity requires not only monitoring the weight of the load, but also controlling the velocity of the movement. Therefore, for this study a load of no more than 57 N (5.82 kg) or a velocity no slower than 73% of maximum swimming velocity was the optimal load range for developing UDK performance, and after increasing the foot area, this interval was expanded to 69 N load (7 kg) or 71% of maximum swimming velocity.

## Conclusions

Athletes can improve their semi-traction swimming training by wearing fins. The F_0_ in the LV relationship can be used as a load criterion for developing athlete's individual in-water resistance training, regardless of whether the athlete wears fins or not. We recommend that athletes use semi-traction swimming to improve UDK performance with a maximum load of no more than 57 N or a velocity of no less than 73% of maximum velocity; wearing fins allows this range to be extended to 69 N and 71% of maximum velocity.
